# Use of dual-mobility cup in primary total hip arthroplasties: an Italian regional register (RIPO) study on three thousand, seven hundred and ten cases

**DOI:** 10.1007/s00264-022-05639-z

**Published:** 2022-11-30

**Authors:** Domenico Tigani, Emanuela Castiello, Alessandro Moghnie, Alessandro Bruschi, Margherita Serra, Luca Amendola, Barbara Bordini

**Affiliations:** 1 Department of Orthopaedic Surgery, Ospedale Maggiore “Carlo Alberto Pizzardi”, Largo Nigrisoli 2, 40133 Bologna, Italy; 2 Department of Biomedical and Neuromotor Science-DIBINEM, IRCCS Rizzoli Orthopaedic Institute, University of Bologna, Via Zamboni 33, 40125 Bologna, Italy; 3 Medical Technology Laboratory, IRCCS – Rizzoli Orthopaedic Institute, Via Di Barbiano 1/10, 40136 Bologna, Italy

**Keywords:** Dual-mobility cup, Standard cup, Total hip arthroplasty, Aseptic loosening, Dislocation, Intraprosthetic dislocation

## Abstract

**Purpose:**

The purpose of the study was to investigate the outcome of dual-mobility cup (DM) compared with a standard cup (SC) in primary total hip arthroplasty (THA) in the long-term follow-up based on a regional Italian joint registry (RIPO).

**Methods:**

The Registry of Prosthetic Orthopaedic Implant (RIPO) was consulted, looking for all primary THAs implanted from 2000 to 2019. Three thousand seven hundred ten were dual-mobility cup (DM) total hip arthroplasties (THA) and 85.816 were standard cup (SC) THAs, on a total of 89.526 primary THA. Demographics, survival rates and causes of revision were evaluated and compared between the two groups.

**Results:**

The use of DM progressively increased from 0.4% in 2000 to 7.5% in 2018 of all primary THAs. Revision rate was 3.5% (128 on 3710) for DMC and 4.7% (4061 on 85,816) for SC. DM presented lower dislocation rate if compared to SC with 22–28-mm femoral head diameter. However, DM showed a higher risk of revision for any causes than SC with 32-mm femoral head diameter in long-term follow-up. Nevertheless, no significant difference was measured in terms of demographics and surgical approach for dislocation rate.

**Conclusions:**

The DM cup represents a valid implant solution and has a lower dislocation rate than 22–28-mm SC. A slight increase in the use of DM implants over time was observed in the RIPO. However, a larger population and a longer follow-up are needed to further monitor the survival rate of new-generation DM implants.

## Introduction

Dual-mobility cup (DM) was introduced since the 1970s for reducing risk of dislocation in both primary and revision hip arthroplasties [1].

Hip instability remains the major complication after THA. Thus, there has been a growing interest on DM implants in the prevention and treatment of hip instability.

According to several joint registries, the Registry of Prosthetic Orthopedic Implants (RIPO) in Emilia Romagna (ER, Italy), which had been collecting data since 2000, considered the dislocation as the second cause of revision of THA.

Literature reports dislocation rate from 0.2 to 7% after primary procedures and up to 21% in revision surgery, representing the second reason for revision at any time and the first cause for early re-operation [2]. However, the DM overall survival rate varies among different studies ranges from 81.4 to 96.3% at 15 years, with a dislocation rate between 0 and 1% [3]. Furthermore, the concept of DM in primary THA also reduced the dislocation rate [4], especially in high-risk patients, i.e. patients with neck femoral fracture, elders and with neurological disease [5]. Despite the encouraging results, DM bearings still have a higher revision rate compared to SC implants. Intraprosthetic dislocation and polyethylene wear were the two main causes of the high revision rate of first-generation DM implants.

**Fig. 1:**
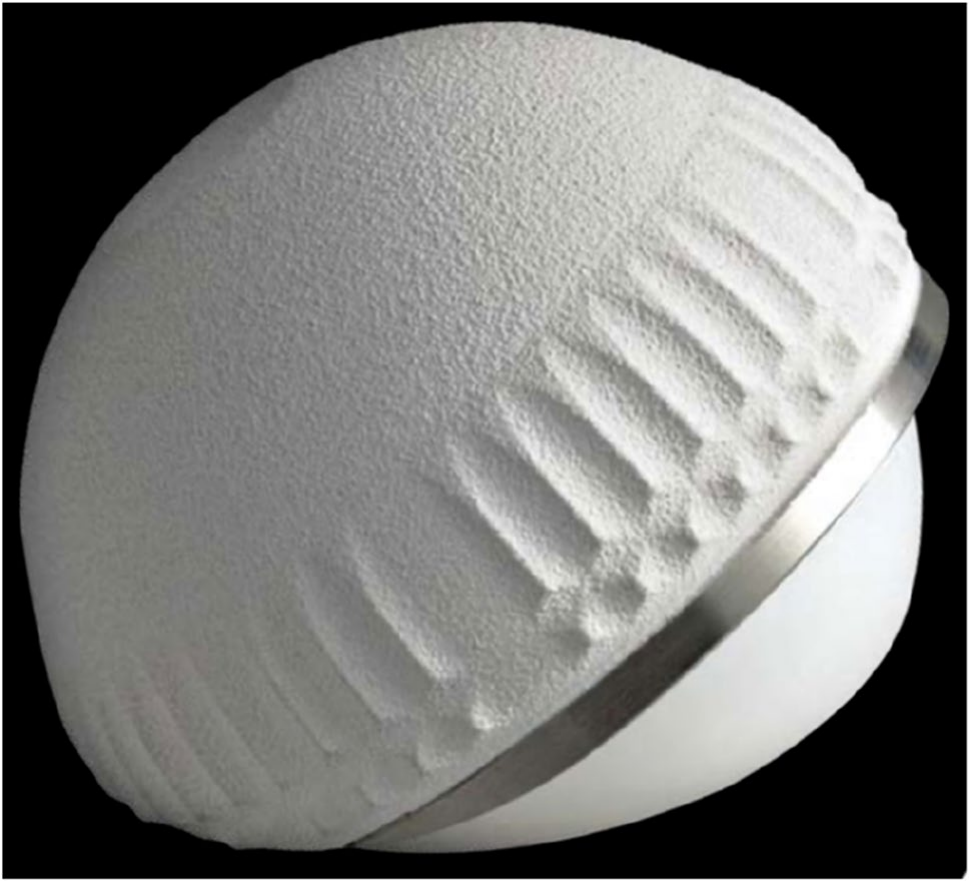
Avantage cup: it was made in stainless steel coated by hydroxyapatite (HA) and later in titanium and HA, being the first press-fit DM commercialized. It was produced also in polished titanium without HA in the cemented version (HA: hydroxyapatite). Courtesy of Biomet Italy

**Fig. 2:**
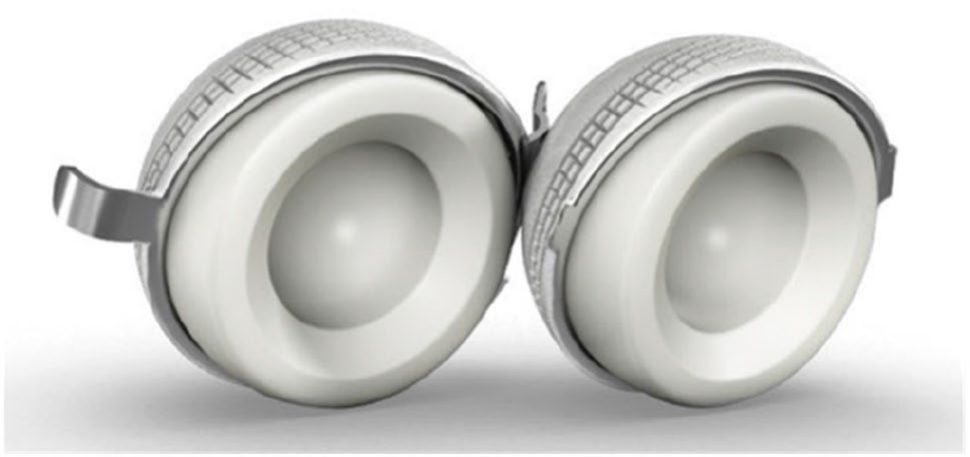
Gyros cup: this implant was made in titanium with HA produced grit blasted or porous coated (HA: hydroxyapatite). Courtesy of Depuy Italy

**Fig. 3:**
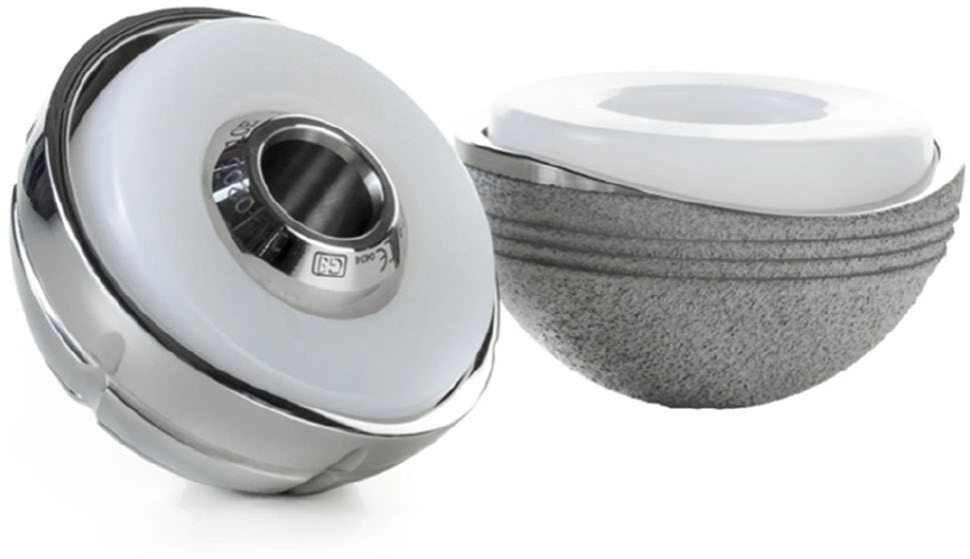
Dualis cup: stainless steel cemented version on the left and steel, HA and titanium made non-cemented version on the right (HA: hydroxyapatite). Courtesy of Gruppo Bioimpianti s.r.l, Italy

**Fig. 4:**
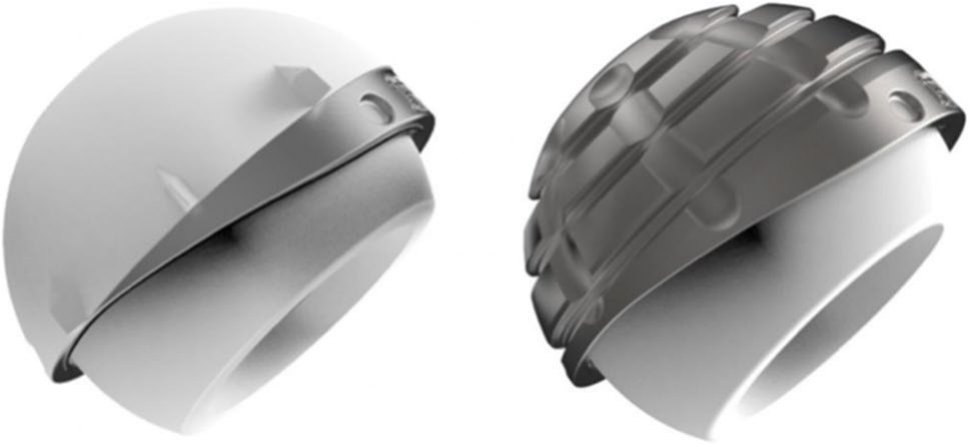
Quattro cup: produced in CrCo, HA and titanium for the non-cemented version (on the left) and without HA for the cemented version (HA: hydroxyapatite). Courtesy of Groupe Lépine Italy

To reduce the revision rate, the DM concept has evolved [6] in its design. Since the first Novae (Serf) cup, many designs have been manufactured with difference in terms of shape, design and surface. The original cup had only a monoblock cementless option, added by the time also cemented as well as modular constructs [7, 8]. Simultaneously, with the production of new systems based on the original Bousquet model, a growing interest was observed and different implant choices have been documented in the period considered. The Avantage cup (Biomet) (Fig. 1) was made in steel coated by hydroxyapatite (HA) and later in titanium and HA, being the first press-fit DM commercialized, produced also in polished steel without HA in the cemented version. On the other side, the EASY cup (the first-generation implant of Novae cup produced by SERF company; in Italy, this acetabular shell, at that time, was distributed by IT Medical company) was made in steel and alumina, whereas the Polarcup (Smith & Nephew) was produced in stainless steel coated by titanium and HA. During the period 2014–2019, the interest of surgeons shifted to the Gyros (Depuy) (Fig. 2), the Dualis (Biompianti) (Fig. 3) and the Quattro arthroplasty (Groupe Lèpine) (Fig. 4). Gyros implant was made in steel with HA produced grit blasted or porous coated [9], while Dualis was steel, HA and titanium made and Quattro was produced in CrCo, HA and titanium [10].

Considering the increasing interest in DM cups in everyday surgical practice, the main purpose of the current study was to analyze the outcome of DM reported in the RIPO from 2000 to 2019. The secondary purpose was to compare data between DMs and SC femoral head diameter (22/28; 32–36) in primary THA in terms of revision rate considering a long-term follow-up, from the Registry of Prosthetic Orthopedic Implants of Emilia Romagna (RIPO).

## Materials and methods

A registry study has been conducted by reporting and analyzing a total of 129,910 primary total hip arthroplasties (THAs) implanted in the period 2000–2019. Data were collected by the Registry of Prosthetic Orthopedic Implants of Emilia Romagna (RIPO), which is a member of the International Society of Arthroplasty Registries. RIPO is a regional population-based register with 4.5 million inhabitants, collecting data about joint arthroplasties performed inside the Emilia Romagna (ER) region, and involving 62 orthopaedic departments with a capture rate of 98%*.* The extraction from the database of our study was made on April 25, 2021.

The inclusion criteria were all primary THAs implanted from 2000 to 2019, which were divided in two groups: dual-mobility cup (DM) THAs and standard cup (SC) THAs, as the control group. Demographics, diagnosis leading to primary implant, articular couplings, head diameters, type of fixation, intra-operative complications, causes of failure and median follow-up time were then collected and compared.

Exclusion criteria were all the THAs procedures performed on patients living outside Emilia Romagna (n.36.228), to minimize bias due to loss to follow-up. Missing data (n.315) and metal-on-metal (MoM) THAs (n.3.841) were also excluded.

Revision was defined as any secondary surgery on the primary THA where one or several components were substituted or explanted.

The primary endpoint was revision of the cup/insert for any cause.

Prosthesis failure is defined as a revision or removal of any prosthesis component and then as the revision or removal of the acetabular/insert component.

Adjustments were made for sex, age at surgery and diameter of the femoral head to discriminate independent risk factors for revision arthroplasty. RIPO collected data as standard practice on all patients, using a format protecting their identity; therefore, approval of the institutional review board was not necessary.

Closed reduction after dislocation without component exchange were not included in RIPO, as in many other registries.

A summary of patients’ and implants characteristics is reported in Table 1.

**Table 1:** Summary of patients’ and implants characteristics

	DM		SC		*p*-value
Sex					*p* = 0.001 (Fisher test)
Male	1356	37.0	33,563	39.1	
Female	2354	63.0	52,253	60.9	
Mean age (min max)	73.1	15–101	68.9	11–100	*p* < 0.001 (*t* test)
Fixation					*p* < 0.001 (chi-square test)
Cemented	84	2.3	4046	4.7	
Hybrid (acetabulum cemented)	152	4.1	436	0.5	
Hybrid (femur cemented)	416	11.2	6886	8.0	
Uncemented	3051	82.2	74,163	86.4	
Missing	7	0.2	285	0.3	
Diagnosis					*p* < 0.001 (chi-square test)
Primary arthritis	1871	50.4	60,654	70.7	
Femoral neck and sequelae	1387	37.4	11,619	13.5	
Other	440	11.9	13,065	15.2	
Missing	12	0.3	478	0.6	
Head diameter (mm)					*p* < 0.001 (chi-square test)
≤ 28	3668	98.9	31,004	36	
32	16	0.4	23,822	28	
≥ 36	26	0.7	30,990	36	
Articular coupling					*p* < 0.001 (chi-square test)
Cer-Cer	4	0.1	35,490	41.4	
Cer-Pol	593	16.0	19,483	22.7	
Met-Pol	1673	45.1	17,018	19.8	
Other	1440	38.8	13,825	16.1	
Surgical approach					*p* < 0.001 (chi-square test)
Anterior	53	1.4	4310	5.1	
Lateral	663	18.0	44,579	52.3	
Posterolateral	2897	78.2	26,562	31.1	
Mini-invasiva	9	0.2	695	0.8	
Other	75	2.0	8698	10.2	
Missing	9	0.2	438	0.5	
BMI					*p* < 0.001 (chi-square test)
Underweight	45	1.2	836	1.0	
Normal	1003	27.0	23,166	27.0	
Overweight	1183	31.9	32,671	38.1	
Obese	675	18.2	15,523	18.1	
Missing	804	21.7	13,620	15.9	

To have a more homogenous population, patients in the SC group were stratified according to head size diameter (22–28, 32, ≥ 36 mm), due to the protective effect of large head against dislocation.

Statistical analyses were performed using SPSS 14.0, version 14.0.1 (SPSS Inc., Chicago, IL) and JMP, version 12.0.1 (SAS Institute Inc., Cary, NC, 1989–2007). Descriptive statistics were used to summarize the data, presented as median and mean with standard deviation (SD) for continuous variables and as frequency with percentage (%) for categorical variables. Statistical significance was calculated using the chi-square test for qualitative data and *t* test for continuous data. A *p* value of < 0.05 was considered statistically significant. Survival curves were calculated and plotted using the Kaplan–Meier method. Cox proportional hazards model was used to investigate the association between the survival time of implants and multiple predictive variables. Adjustments were made for sex, age at surgery, diagnosis at primary THA and diameter of the femoral head to discriminate independent risk factors for revision arthroplasty. Implants were followed until the last date of observation, death of the patient or the end of the study follow-up (December 31, 2019). HR was tested using the Schoenfeld residual method; age at surgery and sex used for adjustment fulfilled the proportional hazard assumption for the whole period. The threshold for significance was *p* = 0.05 for all the tests.

## Results

A total of 129.910 THAs implanted between 1 January 2000 and 31 December 2019 were evaluated. A total of 40.384 THAs were excluded, represented by missing data (*n* = 315), metal-on-metal (MoM) THAs (*n* = 3841) and procedures performed in patients living outside ER (*n* = 36.228), to avoid the bias resulting from the loss to follow-up. 89.526 primary THA met the inclusion criteria.

Among all primary THAs, 3.710 were DM THAs and 85.816 were SC THAs. The median follow-up was 5.1 years (range 0–20) for DM THAs and 7.3 years (range 0–20) for SC THAs.

Of the 3.710 primary DM THAs, 63% were implanted on females and mean age of patients was 73.1 years (range 15–101). 60.9% of primary SC THAs were implanted in females. The most common diagnoses leading to primary THA was primary osteoarthritis in 51% for DM and in 71% for SC. Femoral neck fracture and its complications represented 37% of all primary DM THA, while it was 14% in SC THA.

The vast majority of all THAs was uncemented (86%), posterolateral incision was done in 78% of DM while lateral incision was preferred in SC (52%). Metal polyethylene was implanted in 45% of DM and in 21% of SC, where ceramic-ceramic represented the most bearing chosen (41%).

Twenty-two to twenty-eight-millimeter head diameter was implanted in 99% of DM, while only 37% of SC presented with this component.

The use of DM in primary THAs increased from 15 implants (0.5% of all THAs) in 2000 to 286 (4.8%) in 2019 with more than 20 different types of DM implanted.

DM THAs showed higher intra-operative complication rate when compared to SC THAs (1.6% for DM; 1.4% for SC) without a significant statistical difference.

During the period 2000–2013, a total of 1.737 DMs were implanted, represented by 736 (33.3%) Avantage and 281 (16.2%) EASY cup.

In the period 2014–2019, the most implanted DM cups were Gyros (18.6%), Dualis (14.9%) and Quattro (14.9%).

Dual-mobility cups in primary total hip arthroplasties survival rate was 96.9% (96.2–97.5) at 5 years with 1.532 prostheses at risk and 88.8% (84.2–92.1) at 15 years with 73 prostheses at risk.

SC in primary total hip arthroplasties survival rate was 96.8% (96.7–97.0) at 5 years with 52.104 prostheses at risk and 91.0% (90.6–91.4) at 15 years with 8.397 prostheses at risk.

**Fig. 5:**
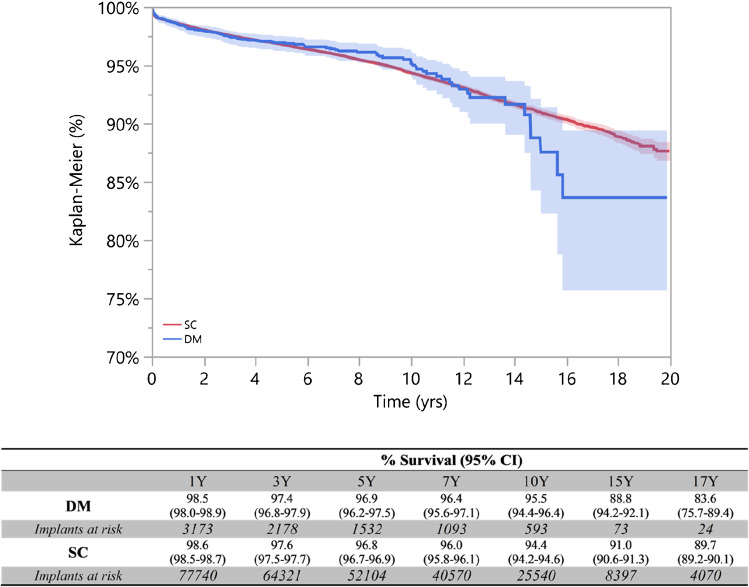
Survival curve of standard cup THA (SC, red line) and dual-mobility cup THA (DM, blue line) (CI 95%) at long-term follow-up

Of the 3.710 DM implants described in this study, 128 failed during the follow-up period and 4061 of 85.816 SC implants. The survival rates of the two cohorts were not statistically different (log-rank test *p* = 0.967) (Fig. 5).

No data available about conservative treatment due to dislocation were recorded.

During the study period, the recurrent prosthesis dislocation occurred in 7 DM (0.2% of the total) and in 578 of 85,816 hips (0.7%) for SC; this value was not statistically significant.

Multivariable Cox regression analysis for DM THA and SC THA showed that SC with head diameter of 22–28 mm was associated with a higher risk of revision due to dislocation than the DM with HR = 1.6 (1.4–2.1), whereas with bigger heads the difference between SC and DM was not significant.

Nevertheless, DM had a higher risk of revision for any causes than SC 32-mm femoral head diameter in the long-term follow-up.

## Discussion

The Registry of Prosthetic Orthopedic Implants of Emilia Romagna (RIPO) reported an increased use of DM for primary THA throughout years.

### Patients’ characteristics

Some differences in patient characteristics have also been observed between patients treated with DM and the ones treated with SC. The RIPO database indicates that in our region patients receiving DM were older than patients receiving SC. Moreover, fractures and their complications count for about 40% of indications for DM, while only 50% of patients treated with DM had a diagnosis of primary osteoarthritis.

Finally, 70% of patients in whom DM was implanted underwent operation through posterolateral approach. All these observations are in line with Literature. Older age, fracture and posterolateral approach are conditions more prone to dislocation and could take advantage of DM [11–13]. Other patient-related factors influencing THA stability, as dementia, neurological conditions and ASA score [14] were not collected, so we were not able to correlate them with choice of implant.

### Implant choice

Different implant choices have been documented in the period considered. During the period 2000–2013, a total of 1.737 DM was registered. The Avantage cup (Biomet) was used in 736 cases, whereas the EASY cup and the Polarcup (Smith&Nephew) were used in 313 and 228 patients respectively. The Avantage cup was made in stainless steel coated by hydroxyapatite (HA) and later in titanium and HA, being the first press-fit DM commercialized, produced also in polished titanium without HA in the cemented version. On the other side, the EASY cup was made in steel and alumina, whereas the Polarcup (Smith&Nephew) was produced in titanium and HA. On the contrary, during the second part (2014–2019), the interest of surgeons shifted to the Gyros (Depuy) counting for 19% of all DMs, the Dualis (Biompianti) 14.9% and the Quattro arthroplasty (Groupe Lèpine) 14.9%. Gyros implant was made in titanium with HA produced grit blasted or porous coated [9], while Dualis was steel, HA and titanium made and Quattro was produced in CrCo, HA and titanium [10].

### Cup fixation and aseptic loosening

A great percentage of the implant were cementless (93.6%). Cemented cups were mainly implanted in the period 2000–2013. The choice of using more cementless implants in the last years was related to the improved design and superficial coating of last generation implants. In fact, the cup became hemispherical in shape with flattened top; this design reduces the stress at the bottom of the cup. Moreover, the dual layer of titanium and HA coating the outer surface of most new implants significantly decreased the incidence of loosening. The incidence of acetabular components loosening of the DM group was comparable to the SC group at one, three, five and seven years and proved to be even lower at ten years.

The survival analysis instead showed a higher incidence of mobilization at 15 years of the DM group compared to the SC group. A possible explanation could be related to the manufactory of first-generation DMs. The original Bousquet implant was produced with stainless steel and alumina was sprayed on the convexity of the metallic cup for promoting bone ingrowth [6]. However, this technique proved to have ineffective bone inductive properties as reported by Boyer et al. [15]. In their study, they shown 8.3% rate of aseptic cup loosening after 11 years and 4.1% retentive failure rate after ten years. These failures were related to the inert non-bioactive alumina coating on the surface that did not allow osteointegration but caused the formation of fibrotic tissue instead of new bone formation in many cases [16]. Avantage cup might contribute to long-term failure of DM cup as well, as reported by the Swedish Registry [17]. However, the reason remains unexplained in the Swedish Registry, but in our opinion, it may be linked to the resorption of HA as indicated by in vitro studies [18]; this might be true above all for Avantage first models, in which steel under HA provides no bone growth properties compared to titanium.

### Dislocation

Long-term studies of first-generation DMs have confirmed that they are an excellent solution against dislocation. Boyer et al. observed no dislocations at a 22-year follow-up on 240 DMs [16]. During the study period, recurrent prosthesis dislocation occurred in seven DM (0.2%) and in 525 of 77.395 SCs (0.68%). This value was non-statistically significant.

We found a statistically significant lower risk for cup revision in the DM THA group compared with SC THA using 22–28-mm femoral head. This is in accordance with other previous studies performed on register data but limiting their analysis to acute neck femoral fracture treatment [19].

Multivariable Cox regression analysis for DM THAs and SC THAs showed that SC THAs with 22–28-mm head diameter were associated with a higher risk of revision due to dislocation than the DM, whereas with bigger heads the difference between SC and DM was not significant. However, DMs had a higher risk for revision, for any causes, than SCs with 32-mm femoral head.

This finding is particularly relevant for two reasons. Firstly, most of the patients receiving DM had higher-than-average risk of post-operative dislocation due to patients’ characteristics of older age and higher percentage indication for fracture treatment if compared to patients in the SC group. Secondly, as reported in other register-based publications [19], revisions of SC THA are often preceded by 1 or more closed reductions (which are not reported in arthroplasty registers), while dislocations of DM THA, intraprosthetic or not, are difficult to treat by closed reduction and more often need surgery with exchange of components, data which are reported in arthroplasty registers.

### Intraprosthetic dislocation (IPD)

In 2001, the surgeon Daniel Noyer pupil of Prof. Gilles Bousquet conducted a study on the mid-term results on DM and he was able to demonstrate the role of the design and the surface finishing of femoral prosthetic neck for the occurrence of the typical complication related to the DM: intraprosthetic dislocation (IPD) [20, 21]. Revisions for IPD, which occurred on average approximately four years after implantation, were twice as likely for rougher necks compared to polished necks. The use of a thin polished neck with a cutaway, chamfered polyethylene liner is recommended to avoid dangerous impingements with femoral neck, suggesting from Noyer himself the term of “third joint” for this area of contact [22].

We did not detect this complication in the RIPO data. In our analysis, none of the implants studied was coupled with Bousquet-type stems. This finding might partially explain our result, although certainly not all femoral necks were smooth and polished. A second explanation could be associated with the almost exclusive use, aside of EASY cup, of second- and third-generation implants.

Nowadays, IPD has almost disappeared due to retentive rim use. Modifications in the design of the liner collar, improvement of proprieties of polyethylene combined with thin trapezoid polished neck, elliptical or circular in shape (introduced progressively with the second generation of DMs at the end of last century and then with the third generation) have probably led to a drastic decrease of this complication [23].

Nowadays, the so-called early dislocations are recently collected by De Martino et al. in a systematic review [23]. According to these authors, most cases have been preceded by an attempted closed reduction in the setting of outer, large articulation dislocation, indicating an iatrogenic aetiology for early IPD.

### Limitations of the study

The principal limitation of our study in common with other registry-based study is related to a possible selection bias. DM was used only in 36 orthopaedics departments among the 62 which contributed to data collection. Moreover, there was the tendency to select DM systems for high-risk elderly patients, especially those with femoral neck fractures, excluding young and active patients.

An additional registry-related limitation is that dislocations treated with closed reduction, without surgical component exchange, were not collected in joint registries. Therefore, the total amount of SC dislocations is unknown and underestimated because of the number of closed reductions which are not reported in the registries. Conversely, DM dislocations are treated with surgery and reported in the database.

A further limitation is that the type of femoral stem was not considered, despite the role of femoral stem involved in the survival of the implant in IPD.

Moreover, our results included mostly the first and the second DM generations, which were implanted in high-risk patients and were correlated to risks of mechanical complications (i.d. loosening, wear, IPD).

Therefore, long-term clinical and registry study are needed to better understand the outcome of the third DM-bearing generation.

## Conclusion

The use of primary DM THA reported in the RIPO had a low long-term complication rate. A lower risk of cup revision due to dislocation in DM THA (*n* = 3.710) compared to SC THA with 22–28 mm femoral head size (*n* = 31.004) (HR = 0.4; CI 0.15–0.64) was found. Furthermore, no statistically significant difference was found, in terms of dislocation, when DM was compared to SC with neither 32-mm or ≥ 36-mm femoral head diameter. Nevertheless, DM had a higher risk of revision for any causes than SC 32-mm femoral head diameter in the long-term follow-up. However, to determine and clarify the exact outcome of DM in primary THA compared with SC, long-term studies with the third-generation DM are needed.

## Data Availability

Data and materials of the present paper are part of the Emilia-Romagna regional register on orthopaedic arthroplasty implantation (RIPO).
